# Increasing the TRPM2 Channel Expression in Human Neuroblastoma SH-SY5Y Cells Augments the Susceptibility to ROS-Induced Cell Death

**DOI:** 10.3390/cells8010028

**Published:** 2019-01-08

**Authors:** Xinfang An, Zixing Fu, Chendi Mai, Weiming Wang, Linyu Wei, Dongliang Li, Chaokun Li, Lin-Hua Jiang

**Affiliations:** 1 Sino-UK Joint Laboratory for Brain Function and Injury and Department of Physiology and Neurobiology, Xinxiang Medical University, Xinxiang 453003, China; anxinfang2018@126.com (X.A.); fuzixing1991@126.com (Z.F.); mcdhsm@163.com (C.M.); wangweiming2015@126.com (W.W.); wly98124@126.com (L.W.); xyldl8@126.com (D.L.); 2 School of Biomedical Sciences, Faculty of Biological Sciences, University of Leeds, Leeds LS2 JT, UK

**Keywords:** human neuroblastoma SH-SY5Y cells, TRPM2 channel, ROS, neuronal cell death

## Abstract

Human neuroblastoma SH-SY5Y cells are a widely-used human neuronal cell model in the study of neurodegeneration. A recent study shows that, 1-methyl-4-phenylpyridine ion (MPP), which selectively causes dopaminergic neuronal death leading to Parkinson’s disease-like symptoms, can reduce SH-SY5Y cell viability by inducing H_2_O_2_ generation and subsequent TRPM2 channel activation. MPP-induced cell death is enhanced by increasing the TRPM2 expression. By contrast, increasing the TRPM2 expression has also been reported to support SH-SY5Y cell survival after exposure to H_2_O_2_, leading to the suggestion of a protective role for the TRPM2 channel. To clarify the role of reactive oxygen species (ROS)-induced TRPM2 channel activation in SH-SY5Y cells, we generated a stable SH-SY5Y cell line overexpressing the human TRPM2 channel and examined cell death and cell viability after exposure to H_2_O_2_ in the wild-type and TRPM2-overexpressing SH-SY5Y cells. Exposure to H_2_O_2_ resulted in concentration-dependent cell death and reduction in cell viability in both cell types. TRPM2 overexpression remarkably augmented H_2_O_2_-induced cell death and reduction in cell viability. Furthermore, H_2_O_2_-induced cell death in both the wild-type and TRPM2-overexpressing cells was prevented by 2-APB, a TRPM2 inhibitor, and also by PJ34 and DPQ, poly(ADP-ribose) polymerase (PARP) inhibitors. Collectively, our results show that increasing the TRPM2 expression renders SH-SY5Y cells to be more susceptible to ROS-induced cell death and reinforce the notion that the TRPM2 channel plays a critical role in conferring ROS-induced cell death. It is anticipated that SH-SY5Y cells can be useful for better understanding the molecular and signaling mechanisms for ROS-induced TRPM2-mediated neurodegeneration in the pathogenesis of neurodegenerative diseases.

## 1. Introduction

Mammalian cells express a large family of transient receptor potential (TRP) cationic channels that are activated by poly-modality and play a role in a diversity of physiological and pathological processes [1,2,3,4,5]. These channels are often grouped based on sequence relatedness into canonical (TRPC), vanilloid (TRPV), melastatin (TRPM), mucolipins (TRPML), polycystins (TRPP) and ankyrin (TRPA) subfamilies. The TRPM2 channel is gated by intracellular ADP-ribose (ADPR) [6] and has been recognised as a key molecular mechanism that confers cells with a prominent sensitivity to reactive oxygen species (ROS), thanks to the well-documented ability of ROS to induce poly(ADPR) polymerase (PARP)-dependent generation of ADPR [7,8,9,10]. The TRPM2 channel is expressed in both excitable and non-excitable cells [7,8,9,10]. More than a decade worth of studies have shown the TRPM2 channel as a common mechanism mediating cell death after exposure to high levels of ROS or diverse pathological stimuli or factors that are known to induce ROS generation. For example, increasing evidence shows that the TRPM2 channel plays an important role in mediating neuronal cell death in the brain, as a result of exposure to ROS, amyloid-β peptide, 1-methyl-4-phenylpyridine ion (MPP) and ischemia-reperfusion [11,12,13,14,15,16,17,18,19,20,21,22,23,24]. Therefore, ROS-induced TRPM2 channel activation has been proposed to contribute to the pathogenesis of ischemia stroke and neurodegenerative conditions such as Alzheimer’s disease (AD) and Parkinson’s diseases (PD) [25,26,27,28,29,30].

Human neuroblastoma SH-SY5Y cells have been widely used as a human neuronal cell model in the study of the molecular and signaling mechanisms for neurodegeneration, particularly those related to PD because of the catecholaminergic, albeit strictly speaking not dopaminergic, neuronal properties [31]. Studies from other groups and us have consistently documented functional expression of the TRPM2 channel in SH-SY5Y cells [21,23,24,32,33,34], but the findings regarding the role of the TRPM2 channel in SH-SY5Y cells are intriguing. A recent study using the 3-(4,5-dimethylthiazol-2-yl)-2,5-diphenyltetrazolium (MTT) assay has shown that concentration-dependent reduction in cell viability after exposure to 10 to 400 µM H_2_O_2_ or 125 to 500 µM MPP via inducing H_2_O_2_ generation [23]. The cytotoxicity induced by both H_2_O_2_ and MPP was significantly suppressed by inhibition of the TRPM2 channel with flufenamic acid (FFA), a TRPM2 channel inhibitor [7]. In addition, MPP-induced detrimental effect on the cell viability was attenuated by small inference RNA (siRNA)-mediated knockdown of the TRPM2 expression and, conversely, enhanced by increasing the TRPM2 expression [23]. These results strongly support that the TRPM2 channel has a critical role in mediating H_2_O_2_/MPP-induced SH-SY5Y cell death [23]. However, an earlier study using the MTT assay showed that increasing the TRPM2 expression in SH-SY5Y cells resulted in higher cell viability after exposure to 50 to 100 µM H_2_O_2_ for 6 or 24 h [32], leading to the recent proposal of a protective role for the TRPM2 channel in cell survival [33,34].

To clarify the role of ROS-induced TRPM2 channel activation in SH-SY5Y cells is critical for the question of whether or not this human cell model can be used to gain insights into the molecular and signaling mechanisms for TRPM2-dependent neurodegeneration. For example, in addition to the revelation of a role for the TRPM2 channel in mediating MPP-induced SH-SY5Y cell death, the recent study by Sun et al. [23] has found strong up-regulation of the TRPM2 expression in MPP-treated SH-SY5Y cells as observed in the substantia nigra pars compacta (SNpc) of the brains from PD patients and PD mice induced by injection with 1-methyl-4-phenyl-tetrahydropyridine to selectively destroy dopaminergic neurons in the SNpc region. These observations raise an interesting question with respect to the role of the TRPM2 channel in mediating dopaminergic neuronal death and, therefore, the pathogenesis of PD. In the present study, we aimed to test the hypothesis that increasing the TRPM2 expression results in greater susceptibility of SH-SY5Y cells to ROS-induced cell death. We generated a stable SH-SY5Y cell line overexpressing the human TRPM2 channel and used the propidium iodide (PI) staining assay to directly examine H_2_O_2_-induced cell death in the wild-type (WT) and TRPM2-overexpressing cells. To make our results more comparable to those reported by previous studies, we also determined cell viability after exposure to H_2_O_2_ in the WT and TRPM2-overexpressing cells. Our results from measuring cell death and cell viability provide consistent evidence to show that exposure to H_2_O_2_ induces SH-SY5Y cell death, regardless of the TRPM2 expression level, and that increasing the TRPM2 expression augments the susceptibility to ROS-induced cell death.

## 2. Materials and Methods

### 2.1. General Chemicals and Culture Medium

General chemicals used in this study were purchased from Sigma, except those indicated specifically. Dulbecco’s modified Eagle’s medium (DMEM) and penicillin/streptomycin were from GIBCO. Foetal bovine serum (FBS) and trypsin-ethylenediaminetetraacetic acid (EDTA) were from Beyotime Biotechnology (Nantong, China).

### 2.2. Cell Culturing

SH-SY5Y cells were kindly provided by Dr. J.A. Sim (University of Manchester, Manchester, UK) and maintained in standard cell culture medium (DMEM supplemented with 10% FBS, 100 units/mL of penicillin and 100 µg/mL of streptomycin) in a tissue incubator (Thermofisher, Waltham, MA, USA) at 37 °C in the presence of 5% CO_2_. Cells were passaged every 3 to 4 days or when they became 70% to 80% confluent.

### 2.3. Propidium Iodide (PI) Staining Cell Death Assay

Cell death was examined using the PI staining assay as described in our previous studies [24,35]. In brief, cells were seeded in 24-well plates at 2 × 10^4^ cells per well in 500 μL of standard culture medium and incubated overnight. After cells were treated with H_2_O_2_, PI, and Hoechst 33342 were added into the culture medium with the final concentrations of 2 μg/mL and 1 μg/mL, respectively. Cells were incubated at 37 °C for 30 min. In experiments examining the effects of inhibitors on H_2_O_2_-induced cell death, cells were treated with the inhibitor at indicated concentrations 30 min before and during exposure to H_2_O_2_. Images were captured using an AxioVert A1 fluorescence microscope (Zeiss). For each condition in every independent experiment, three wells of cells were used, one randomly selected field in each well was imaged, and at least 500 cells from three images/wells were analyzed. Cell death was presented as a percentage of PI-positive cells in all cells identified by Hoechst 33342 staining in the same fields.

### 2.4. Generation of SH-SY5Y Cells Overexpressing the Human TRPM2 Channel

The internal ribosome entry site (IRES) bicistronic construct that enables expression of separate TRPM2 protein and enhanced green fluorescent protein (GFP) under the control of the same promoter, pTRPM2-IRES-GFP, was generated by ligation of the full-length human TRPM2 cDNA [6] and pGFP-IRES fragment that were both amplified by polymerase chain reaction (PCR). Primers for PCR are available upon request. PCR was performed using Phusion polymerase (New England Biolabs, Beijing, China) and the following protocols: 120 s at 98 °C, followed by 25 cycles for TRPM2 or 20 cycles for pGFP-IRES fragment, and a final step of 10 min at 72 °C. Each cycle comprised 30 s at 98 °C, 30 s at 58 °C, and 5 min at 72 °C for TRPM2 or 30 s at 98 °C, 30 s at 58 °C, and 3 min at 72 °C for pGFP-IRES. Ligated PCR products were transformed into competent DH5α *Escherichia coli* (Takara, Beijing, China). Positive colonies were identified by PCR and sequencing the PCR products. Plasmids were purified, and the construct was further confirmed by DNA sequencing.

For transfection, SH-SY5Y cells were cultured in standard culture medium in six-well plates and transfected with the pTRPM2-IRES-GFP construct using Xfect transfection reagent (Clonetech, Beijing, China) according to the manufacturer’s instructions. Transfected cells were identified by examining GFP expression using a fluorescence microscope 48 h post transfection and also using flow cytometry 72 h post transfection. GFP-positive SH-SY5Y cells were incubated in standard culture medium containing 400 μg/mL G418 for another 2 weeks. Individual cells were inoculated into a 96-well plate and, after being cultured in G418-containing standard culture medium for 1 month, cells showing strong GFP expression were selected and expanded. The TRPM2 protein expression in the stable cell line used in this study was further verified by Western blotting.

### 2.5. Flow Cytometry

Cells were cultured for 3 days before they were harvested for analysis using flow cytometry. Approximately 10,000 cells were analyzed for positive expression of GFP, using a flow cytometer (BD Biosciences, Beijing, China) and 488 nm/512 nm filters.

### 2.6. Western Blotting

The TRPM2 protein expression was examined using standard sodium dodecyl sulfate polyacrylamide gel electrophoresis (SDS-PAGE) and Western blotting. In brief, cell lysates were prepared in the radioimmuno-precipitation assay buffer (Beyotime Biotechnology, Nantong, China) containing 1 mM phenylmethane sulfonyl fluoride. The protein concentrations in cell lysates were determined using a bicinchoninic acid assay kit (Beyotime Biotechnology). Twenty microliters of cell lysate containing 80 µg proteins alongside protein markers (Beyotime Biotechnology) were separated by electrophoresis on 15% SDS-PAGE gels and transferred to nitrocellular membranes (Millipore, Burlington, MA, USA). The membranes were blocked by 5% non-fat milk in Tris-buffered saline containing 0.05% Tween 20 (TBST) and then incubated with the primary anti-TRPM2 antibody at a dilution of 1:200 (ab87050, Abcam, Shanghai, China) or anti-glyceraldehyde 3-phosphate dehydrogenase (GAPDH) at 1:1000 (Hangzhou Goodhere Biotechnology Co, Hangzhou, China) at 4 °C overnight. After extensive washing in TBST, the membranes were incubated with the secondary horseradish peroxidase-conjugated goat anti-rabbit IgG antibody at 1:800 (Affinity Biosciences, Cincinnati, OH, USA) at room temperature for 1 h. After extensive washing in TBST, the proteins were visualized using an enhanced chemiluminescence kit (Beyotime Biotechnology), and the images were captured using an Amersham Imager 600 system (GE Healthcare, Chicago, IL, USA).

### 2.7. Cell Counting Kit-8 (CCK-8) Cell Viability Assay

The cell viability was examined using Cell Counting Kit-8 (CCK-8) assay kits (Dojindo Molecular Technologies, Shanghai, China) according to the manufacturer’s instructions. Cells were seeded in 96-cell plates at 1 × 10^4^ cells per well in 100 μL of standard culture medium and incubated overnight. After cells were treated with H_2_O_2_ at indicated concentrations, 10 μL of CCK-8 reagent was added to each well and incubated in a tissue culture incubator at 37 °C for 1 h. The absorbance at 450 nm was determined, using a Bio-Tek800 microplate reader (BioTek Instruments, Winooski, VT, USA). Three wells of cells were used for each condition for every independent experiment. The cell viability in H_2_O_2_-treated cells was presented as a percentage of that in control or untreated cells in parallel experiments.

### 2.8. Data Presentation and Statistical Analysis

The results are presented as mean ± standard error mean (SEM), where appropriate, with “n” representing the number of independent experiments. Comparisons were performed using GraphPad Prism software, with *p* < 0.05 considered to be statistically significant. One-way analysis of variance (ANOVA) and post hoc Tukey’s test were used for multiple groups to compare cell death or cell viability for the same type of cells, that is, WT or TRPM2-overexpressing cells. Two-way ANOVA and post hoc Tukey’s test were used to compare cell death or cell viability between the WT and TRPM2-overexpressing cells that were treated with the same concentration of H_2_O_2_.

## 3. Results

### 3.1. H_2_O_2_ Induces Cell Death via PARP-Dependent TRPM2 Channel Activation

**Figure 1:**
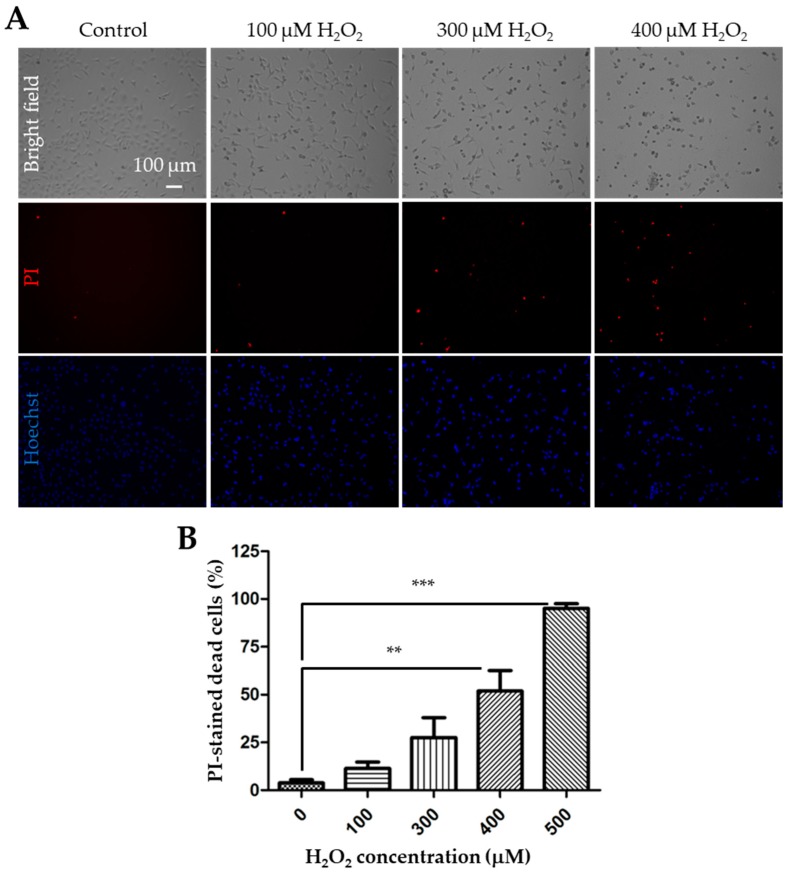
Exposure to H_2_O_2_ induces concentration-dependent cell death in SH-SY5Y cells. (**A**) Representative bright field and fluorescence images showing propidium iodide (PI) and Hoechst staining in control cells and cells after exposure to 100, 300 or 400 μM H_2_O_2_ for 24 h. (**B**) Mean ± standard error mean (SEM) percentage of cell death after exposure to indicated concentrations of H_2_O_2_ from 3 independent experiments. **, *p* < 0.01 and ***, *p* < 0.001, compared to control cells without exposure to H_2_O_2_.

We started with using the PI staining assay to detect cell death following exposure to 100 to 500 μM H_2_O_2_ for 24 h in the WT SH-SY5Y cells. Representative images are shown in Figure 1A, and the results from three independent experiments are summarized in Figure 1B. Exposure to H_2_O_2_ resulted in concentration-dependent cell death. The cell death was negligible (3.8 ± 1.0%, n = 3) under control conditions or in untreated cells, but there was a noticeable increase in cell death after exposure to H_2_O_2_, with 11.4 ± 2.0%, 27.6 ± 6.0%, 51.9 ± 6.2%, and 95.0 ± 1.4% at 100, 300, 400, and 500 µM H_2_O_2_, respectively, although the increase reached a statistically significant level (*p* < 0.05) only for 400 and 500 µM H_2_O_2_.

**Figure 2:**
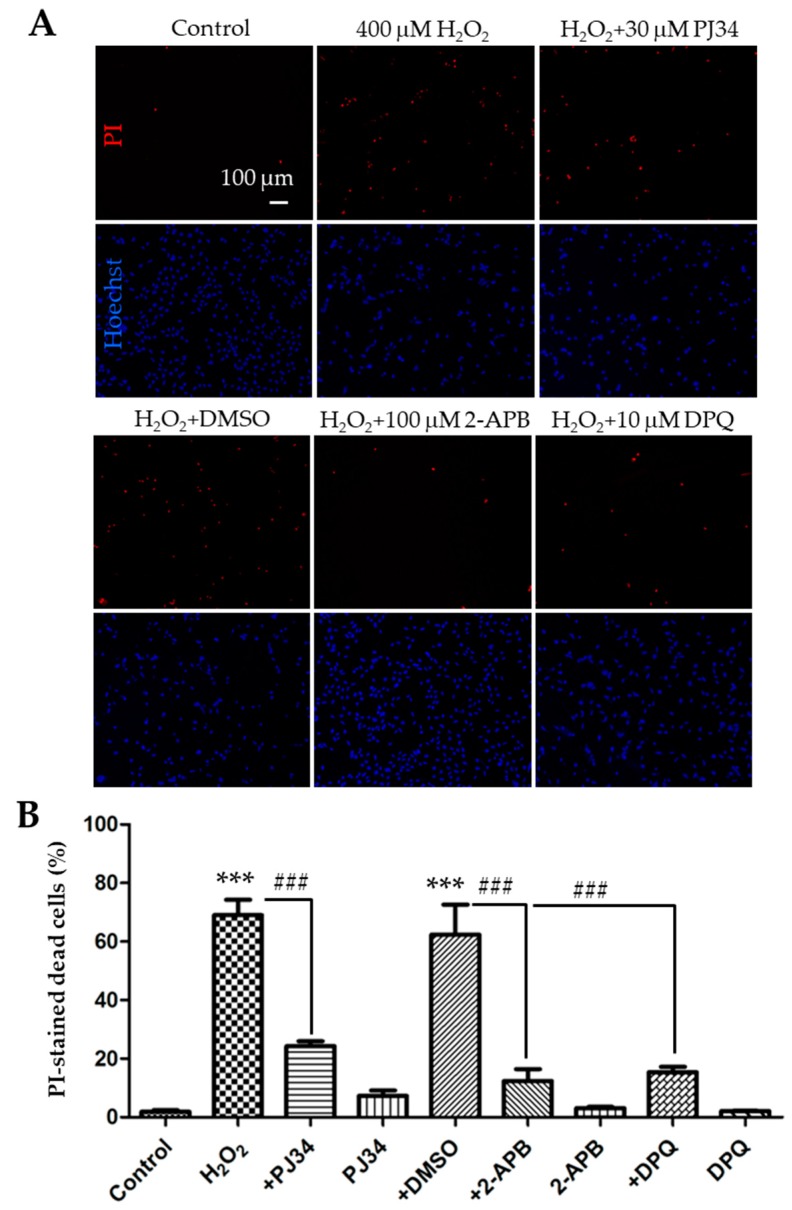
Effects of treatment with polymerase (PARP) or melastatin 2 (TRPM2) channel inhibitor on H_2_O_2_-induced cell death in SH-SY5Y cells. (**A**) Representative fluorescence images showing staining with PI and Hoechst of control cells and cells treated with indicated conditions. Treatment with the inhibitors or dimethyl sulfoxide (DMSO) started 30 min before and during exposure to 400 μM H_2_O_2_ for 24 h. (**B**) Mean ± SEM percentage of PI-positive cells under indicated conditions, as shown in panel A, from three independent experiments. ***, *p* < 0.001 compared with untreated cells, and ###, *p* < 0.001 compared to cells treated with H_2_O_2_ alone or treated with H_2_O_2_ and DMSO.

As has been shown in many cell types, ROS can activate the TRPM2 channel via a PARP-dependent mechanism [7]. To confirm that H_2_O_2_-induced cell death resulted from PARP-dependent TRPM2 channel activation in SH-SY5Y cells, we moved on to examine the effects of pharmacological inhibitors of PARP. Cells were treated 30 min before and during 24 h exposure to 400 µM H_2_O_2_ with 30 µM PJ34 and 10 µM 3,4-dihydro-5[4-(1-piperindinyl)butoxy]-1(2H)-isoquinoline (DPQ), two structurally distinct PARP inhibitors that have been shown in our recent study to be effective in inhibiting H_2_O_2_-induced TRPM2-mediated microglial cell death [34]. Indeed, cell death was reduced from 69.1 ± 3.0% in cells treated with H_2_O_2_ alone to 24.3 ± 1.0% in cells that were also treated with PJ34, indicating a strong inhibition (Figure 2A,B; n = 3; *p < 0*.001). Similarly, H_2_O_2_-induced cell death was reduced to 15.4 ± 1.1% in DPQ-treated cells from 62.3 ± 5.9% in cells treated with dimethyl sulfoxide (DMSO), the solvent used to prepare DPQ stock solution (Figure 2A,B; n = 3; *p* < 0.001). We next examined the effect of treatment with 100 µM 2-APB, a TRPM2 channel blocker that can inhibit H_2_O_2_-induced TRPM2-mediated cell death [34]. Treatment with 2-APB also strongly suppressed H_2_O_2_-induced cell death from 62.3 ± 5.9% in DMSO-treated cells to 12.4 ± 2.4% in 2-APB-treated cells (Figure 2A,B; n = 3; *p* < 0.001). Treatment with DMSO or the inhibitor alone resulted in no or minimal increase in cell death (Figure 2B). Collectively, these results provide evidence to support the notion that PARP-dependent TRPM2 channel activation mediates H_2_O_2_-induced cell death in SH-SY5Y cells, which is consistent with a recent study that reports a role for the TRPM2 channel in H_2_O_2_-induced cell death in SH-SY5Y cells based on the measurement of cell viability [23].

### 3.2. TRPM2 Overexpression Augments the Susceptibility to H_2_O_2_-Induced Cell Death

**Figure 3:**
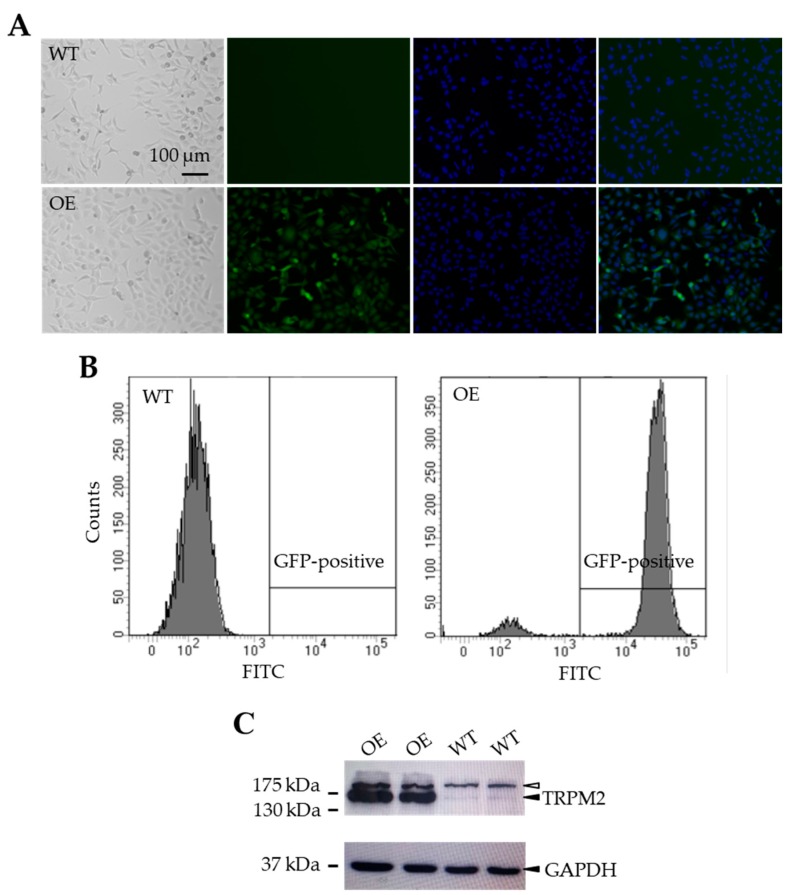
Generation of TRPM2-overexpressing SH-SY5Y stable cells. (**A**) Represent bright field and fluorescence images of wild-type (WT) and TRPM2-overexpressing (OE) cells. (**B**) Flow cytometry analysis of green fluorescent protein (GFP) expression in the WT (left) and OE (right) cells. (**C**) Western blots showing expression of TRPM2 and glyceraldehyde 3-phosphate dehydrogenase (GAPDH) in the WT and OE cells. The solid arrowheads in the top and bottom panels indicate TRPM2 and GAPDH, respectively. The empty arrowhead in the top panel indicates a non-specific and high molecular weight protein, which was present in both the WT and OE cells with a similar level. There was a strong increase in the TRPM2 protein expression in the OE cells (lanes 1–2) as compared to the endogenous TRPM2 protein expression in the WT cells (lanes 3–4), where the GAPDH expression level was similar in both cell types.

To examine the effect of increasing the TRPM2 expression in SH-SY5Y cells on H_2_O_2_-induced cell death, we made a mammalian expression construct using the IRES technology to generate a stable SH-SY5Y cell line expressing the human TRPM2 protein and GFP under the control of the same promoter (see Materials and Methods). Positively transfected SH-SY5Y cells were initially identified by the positive GFP expression as shown using fluorescent microscopic imaging (Figure 3A) and flow cytometry analysis (Figure 3B). As shown by Western blotting, SH-SY5Y cells showed endogenous expression of the TRPM2 protein with the expected molecular weight (171 kDa), as reported in previous studies [23,24] and, as anticipated, the TRPM2 protein expression was noticeably increased in the GFP-positive cells (Figure 3C).

**Figure 4:**
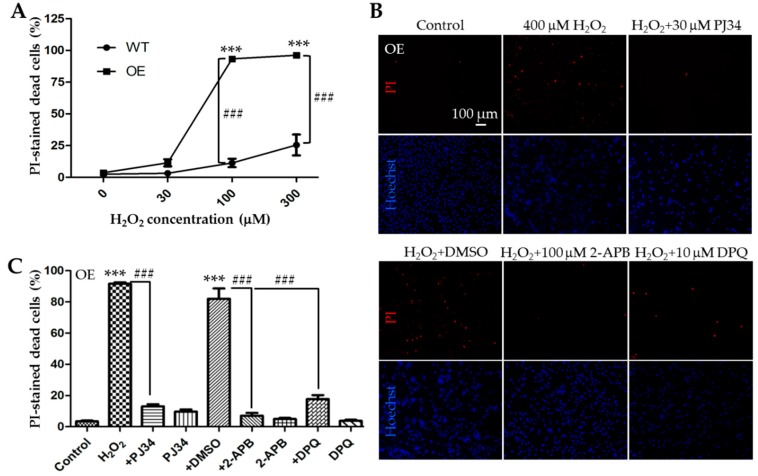
Increasing the TRPM2 expression in SH-SY5Y cells augments the susceptibility to H_2_O_2_-induced cell death. (**A**) Mean ± SEM percentage of PI-positive cells in the WT and TRPM2-overexpressing (OE) cells after exposure to indicated concentrations of H_2_O_2_ from three independent experiments. ***, *p* < 0.001, compared to cells without exposure to H_2_O_2_ in the OE cells; ###, *p* < 0.001 compared between the WT and OE cells exposed to the same concentration of H_2_O_2_. (**B**) Representative fluorescence images showing staining with PI and Hoechst in the OE cells treated with indicated inhibitors, 30 min before and during exposure to 400 μM H_2_O_2_ for 24 h. (**C**) Mean ± SEM percentage of PI-positive cells under indicated conditions in the OE cells from three independent experiments. ***, *p* < 0.001 compared to control cells; ###, *p* < 0.001 compared to cells treated with H_2_O_2_ alone or treated with H_2_O_2_ and DMSO.

We next performed parallel experiments using the PI staining assay to compare H_2_O_2_-induced cell death in the WT and TRPM2-overexpressing SH-SY5Y cells after exposure to 30, 100, and 300 µM H_2_O_2_ for 24 h. The results from three independent experiments are summarized in Figure 4A. After exposure to 30 µM H_2_O_2_, there was a slight but statistically insignificant increase in cell death in the TRPM2-overexpressing cells as compared to that in the WT cells. Cell death upon exposure to 100 μM H_2_O_2_ was increased from 11.3 ± 1.9% in the WT cells to 93.3 ± 1.0% in the TRPM2-overexpressing cells (Figure 4A; n = 3; *p* < 0.001). Similarly, cell death induced by exposure to 300 μM H_2_O_2_ was increased from 25.5 ± 4.8% in the WT cells to 96.2 ± 0.4% in the TRPM2-overexpressing cells (Figure 4A; n = 3; *p* < 0.001). These results clearly indicate that increasing the TRPM2 expression in SH-SY5Y cells remarkably augments the susceptibility to H_2_O_2_-induced cell death. To verify that such increased H_2_O_2_-induced cell death was mediated by PARP-dependent activation of the TRPM2 channel as shown above in the WT cells, we examined the effects of PJ34, DPQ or 2-APB on H_2_O_2_-induced cell death in the TRPM2-overexpressing cells. We used the same experimental conditions for the TRPM2-overexpressing cell as for the WT cells described above, that is, exposure of cells to 400 µM H_2_O_2_ for 24 h and treatment of cells with 30 µM PJ34, 10 µM DPQ, or 100 µM 2-APB 30 min before and during exposure to H_2_O_2_. The results are shown in Figure 4B,C. Cell death in the TRPM2-overexpressing cells was reduced from 91.7 ± 0.5% in cells treated with H_2_O_2_ alone to 13.1 ± 0.8% (n = 3; *p* < 0.001) in cells that were also treated with PJ34. Similarly, H_2_O_2_-induced cell death of 82.0 ± 3.8% in DMSO-treated cells was reduced to 17.7 ± 1.5% (n = 3; *p* < 0.001) in DPQ-treated cells or 7.1 ± 1.0% in 2-APB-treated cells (n = 3; *p* < 0.001). Again, treatment with DMSO or the inhibitor alone caused no or minimal cell death in the TRPM2-overexpressing cells (Figure 4C). These pharmacological results are readily reconciled with the above-described observation that increasing the TRPM2 expression enhanced the susceptibility to ROS-induced cell death and, taken together, provide consistent evidence to support that ROS-induced PARP-dependent activation of the TRPM2 channel, regardless of its expression, results in cell death.

### 3.3. TRPM2 Overexpression Reduces the Cell Viability after Exposure to H_2_O_2_

**Figure 5:**
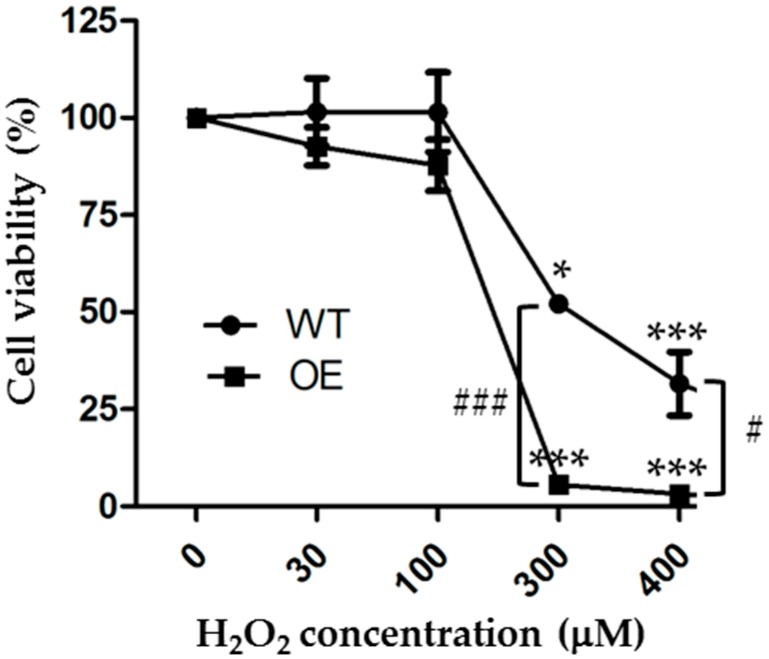
Increasing the TRPM2 expression in SH-SY5Y cell enhances H_2_O_2_-induced reduction in cell viability. Mean ± SEM cell viability, as a percentage of that under control conditions, in the WT and TRPM2-overexpressing (OE) cells after exposure to indicated concentrations of H_2_O_2_ for 24 h. The results were from three independent experiments. *, *p* < 0.05 and ***, *p* < 0.001, compared to cells without exposure to H_2_O_2_ in the WT or OE cells, respectively; #, *p* < 0.05 and ###, *p* < 0.001 compared between the WT and OE cells treated with the same concentration of H_2_O_2_.

As mentioned above, previous studies mainly used the MTT assay to examine the cell viability and the effects of increasing the TRPM2 expression on the cell viability after exposure to H_2_O_2_ or MPP [23,32]. The MTT assay is a metabolic assay that measures the live cells, whereas the PI assay is used to detect cell death, particularly necrotic cell death. To make more direct or meaningful comparisons, we finally examined the effects of exposure to 30 to 400 µM H_2_O_2_ for 24 h on the cell viability of the WT and TRPM2-overexpressing SH-SY5Y cells using the CCK-8 assay, which is based on the same principle as the MTT assay. Our results from three independent experiments are summarized in Figure 5. Exposure to H_2_O_2_ resulted in a concentration-dependent reduction in cell viability in both the WT and TRPM2-overexpressing cells. In the WT cells, the mean cell viability was 101.5 ± 5.0%, 101.5 ± 5.9%, 52.1 ± 0.6%, and 31.6 ± 4.7% after 24 h exposure to 30, 100, 300, and 400 µM H_2_O_2_, respectively. The cell viability was statistically significantly lower after exposure to 300 µM (*p* < 0.05) or 400 µM H_2_O_2_ (*p* < 0.001), but not 30 or 100 µM H_2_O_2_. These results are similar to those in a recent study reporting a significant reduction in cell viability after exposure to 200 or 400 µM, but not 10 or 100 µM H_2_O_2_ [23]. In the TRPM2-overexpressing cells, the mean cell viability was 92.6 ± 2.8%, 87.8 ± 3.8%, 5.6 ± 0.4%, and 3.2 ± 0.2% after exposure to 30, 100, 300, and 400 µM H_2_O_2_, respectively. The cell viability was significantly lower after exposure to 300 µM or 400 µM H_2_O_2_ (*p* < 0.001 in both cases), but not 30 or 100 µM H_2_O_2_ (Figure 5). The mean cell viability in the TRPM2-overexpressing cells was lower compared to that in the WT cells after exposure to the same concentration of H_2_O_2_, but the difference reached a statistically significant level only for 300 µM (*p* < 0.001) and 400 µM H_2_O_2_ (*p* < 0.05) (Figure 5). These results from measuring cell viability also show that TRPM2 overexpression increases the susceptibility of SH-SY5Y cells to ROS-induced cell death.

## 4. Discussion

The present study shows that exposure of SH-SY5Y cells to H_2_O_2_ induced concentration-dependent cell death as well as confirming the finding that H_2_O_2_ induced a concentration-dependent reduction in cell viability. Furthermore, we show that increasing the TRPM2 expression enhanced H_2_O_2_-induced cell death and reduction in cell viability, consistently indicating that increasing the TRPM2 expression in SH-SY5Y cells augments the susceptibility to H_2_O_2_-induced cell death.

The TRPM2 channel has been shown to mediate ROS-induced cell death in many cell types [7]. In SH-SY5Y cells, previous studies using the MTT assay showed a concentration-dependent reduction in cell viability after exposure to 50 to 100 µM H_2_O_2_ [32] or 10 to 400 µM H_2_O_2_ or 125 to 500 µM MPP via inducing H_2_O_2_ generation [23]. The present study also demonstrated concentration-dependent cell death after exposure to 30 to 500 µM H_2_O_2_, using the PI staining assay (Figure 1 and Figure 4A), and also a concentration-dependent reduction in cell viability after exposure to 30 to 400 µM H_2_O_2_, using the CCK-8 assay (Figure 5). However, regarding the effects of H_2_O_2_ on cell viability, there were noticeable differences between the present and previous studies. The earlier study reported that exposure to 50 or 100 µM H_2_O_2_ for 6 or 24 h reduced the cell viability [32]. The present study found a significant reduction in cell viability only after 24 h exposure to higher concentrations of H_2_O_2_ (300 and 400 µM) but not to lower concentrations including 100 µM H_2_O_2_, the condition used in the aforementioned previous study [32]. In accordance with our study, Sun et al. have recently shown that the cell viability was significantly reduced after exposure to 200 and 400 µM H_2_O_2_, but not 10 or 100 µM H_2_O_2_ [23]. They also reported reduced cell viability following exposure to MPP at high concentrations (250 and 500 µM) but not at a low concentration (125 µM) [23]. The reasons for these discrepancies remain unclear but the cells used, particularly the proliferative properties of the cells, are likely to contribute, considering that cell proliferation can influence the number of live cells which is measured using the MTT and CCK-8 assays.

In the present study, we showed significant inhibition of H_2_O_2_-induced cell death by 2-APB (Figure 2). Similarly, Sun et al. have reported suppression by FFA of H_2_O_2_-induced reduction in cell viability [23]. Both 2-APB and FFA are known to inhibit the TRPM2 channel, but neither of them is specific to the TRPM2 channel [7]. Nonetheless, the inhibition of H_2_O_2_-induced cell death by 2-APB in this study and by FFA in the study by Sun et al. [23] are in support of the notion that the TRPM2 channel activation is critical for H_2_O_2_-induced cell death or reduction in cell viability. We also showed that H_2_O_2_-induced cell death was strongly inhibited by PJ34 and DPQ (Figure 2), confirming an important role for PARP in ROS-induced TRPM2 channel activation and ensuing cell death [7]. We showed here that increasing the TRPM2 expression augmented H_2_O_2_-induced reduction in cell viability as well as H_2_O_2_-induced cell death (Figure 4A and Figure 5). Sun et al. have reported that MPP-induced reduction in cell viability was strongly attenuated by FFA and also by siRNA-mediated knockdown of the TRPM2 expression and by contrast exacerbated by increasing the TRPM2 expression [23]. The observations that H_2_O_2_-induced cell death in the TRPM2-overexpressing cells was largely prevented by 2-APB, PJ34, and DPQ (Figure 4B,C) strongly disfavor, albeit not completely ruling out, the possibility that alterations in TRPM2-independent mechanism(s) give rise to the increased susceptibility to H_2_O_2_-induced cell death. Overall, our results suggest that ROS-induced PARP-dependent activation of the TRPM2 channel, regardless of its expression level, leads to SH-SY5Y cell death. These findings reinforce the notion that the TRPM2 channel is a key and common meditator for ROS-induced cell death in SH-SY5Y cells [23,24] as reported in neurons [11,12,13,21] and other types of cells [7,35,36,37,38].

However, studies examining the effect of increasing the TRPM2 expression in SH-SY5Y have led to the proposal of a different role for the TRPM2 channel [32,33,34]. An earlier study reported that the reduction in cell viability after exposure to 50 and 100 µM H_2_O_2_ for 6 or 24 h was attenuated by increasing the TRPM2 expression, suggesting a role for the TRPM2 channel in supporting cell survival and proliferation [32]. As we showed here, increasing the TRPM2 expression resulted in no significant protection against the reduction in cell viability after exposure to 30 and 100 µM H_2_O_2_ for 24 h, but a further reduction in cell viability after exposure to 300 and 400 µM H_2_O_2_ for 24 h (Figure 5). The study by Sun et al., while not examining the effect on H_2_O_2_-induced reduction in cell viability, has shown that increasing the TRPM2 expression enhanced the reduction in cell viability after exposure to a high concentration of MPP [23], as we observed here after exposure to high concentrations of H_2_O_2_. As discussed above, it is highly likely that the discrepancy in part arises from the cells used. It is worth mentioning that increasing the TRPM2 expression has been shown to induce cell death or reduce cell viability after exposure to H_2_O_2_ in other cell types. For example, in human embryonic kidney 293 cells that are largely void of endogenous TRPM2 expression, overexpression of the recombinant TRPM2 channel strongly reduced cell viability following exposure to H_2_O_2_, determined using the trypan blue exclusion assay, or increased cell death after exposure to H_2_O_2_, shown by staining with PI and Alexa Fluor 488-annexin V [36]. Likewise, in human monocytic U937 cells which endogenously express the TRPM2 channel, increasing the TRPM2 expression enhanced H_2_O_2_-induced reduction in cell viability based on the trypan blue exclusion assay and apoptotic cell death determined by staining with Alexa Fluor 594-annexin V [37].

As already mentioned above, cell death has been assessed directly by PI staining for necrotic cell death or annexin V staining for apoptotic cell death and also indirectly using the MTT and CCK-8 assays that measure the number of live cells. In this study, we assessed H_2_O_2_-induce cell death using the PI staining and CCK-8 assays. As discussed above, the results from these two measurements overall provide consistent evidence to show that exposure to H_2_O_2_ resulted in concentration-dependent cell death and that increasing the TRPM2 expression enhanced the susceptibility to H_2_O_2_-induced cell death (Figure 1, Figure 4A, and Figure 5). Nonetheless, a close examination of the data reveals a noticeable difference. Increasing the TRPM2 expression in SH-SY5Y cells conferred a significant increase in cell death (Figure 4A) but resulted in a modest reduction in cell viability after exposure to 100 µM H_2_O_2_ (Figure 5). Such a difference may result from the experimental conditions used (e.g., a high cell seeding density used for the CCK8 assay versus a low cell seeding density for the PI staining assay) as well as the aforementioned proliferative properties of the cells. Therefore, cautions needs to be exercised in conducting quantitative comparisons and interpretation of the results obtained using different experimental conditions and/or methods.

Recent studies, using TRPM2-knockout mice in combination with disease models, support a critical role for the TRPM2 channel in mediating ROS-induced neuronal death and in contributing to the pathogenesis of ischemia-reperfusion brain damage and neurodegenerative diseases, such as AD [27,28,29,30]. SH-SY5Y cells have been useful as a human neuronal cell model in the study of neurodegeneration [31]. In a recent study using SH-SY5Y cells, we have recently revealed TRPM2 channel activation as a critical step in a positive feedback signaling mechanism that causes lysosomal and mitochondrial dysfunction to drive delayed neuronal cell death [24]. Such information is helpful in gaining mechanistic insights into TRPM2-dependent delayed neuronal death responsible for ischemia-reperfusion brain damage [15,18]. As already introduced above, in addition to the revelation of the importance of the TRPM2 channel in mediating MPP-induced SH-SY5Y cell death, Sun et al. have shown strong up-regulation of the TRPM2 expression in MPP-treated SH-SY5Y cells as observed in the SNpc region of the brains from PD patients and also PD mice [23]. These new findings point to the TRPM2 channel for its potential role in mediating loss of dopaminergic neurons, the key event in the pathogenesis of PD [39]. It is anticipated that SH-SY5Y cells should be useful for researchers to gain a better understanding of TRPM2-dependent signaling mechanisms for neurodegeneration that are relevant to the pathogenesis of PD.

## 5. Conclusions

The present study provides evidence to show that PARP-dependent activation of the TRPM2 channel in SH-SY5Y cell mediates ROS-induced cell death and increasing the TRPM2 expression augments the susceptibility to ROS-induced death. As shown by recent studies, using SH-SY5Y cells as a human neuronal cell model should help in interrogating TRPM2-dependent signaling mechanisms in neuronal cell death and related neurodegenerative diseases.
